# Simultaneous determination of five azadirachtins in the seed and leaf extracts of *Azadirachta indica* by automated online solid-phase extraction coupled with LC–Q-TOF–MS

**DOI:** 10.1186/s13065-018-0453-y

**Published:** 2018-07-19

**Authors:** Li Song, Jin Wang, Quan Gao, Xiaojiang Ma, Yuwei Wang, Yaoyao Zhang, Hang Xun, Xi Yao, Feng Tang

**Affiliations:** SFA Key Laboratory of Bamboo and Rattan Science and Technology, International Centre for Bamboo and Rattan, No. 8 Futong Dongdajie, Wangjing, Chaoyang District, Beijing, 100102 China

**Keywords:** *Azadirachta indica*, Neem, Online solid-phase extraction, Azadirachtin, LC–Q-TOF–MS, Method validation

## Abstract

Neem (*Azadirachta indica*) extract is well-known as a natural pesticide for the control of agricultural pests. Azadirachtin A and its structural analogues are considered as active compounds. However, the amounts of azadirachtins varies in neem extracts, providing a variety of insecticidal activities. In this study, a novel method of automated online solid-phase extraction coupled with liquid chromatography/quadrupole-time-of-flight mass spectrometry (SPE-LC–Q-TOF–MS) was developed and validated for simultaneous quantification of five azadirachtins (azadirachtins A, B, D, H and I) in seed and leaf extracts of *A. indica*. Different experimental parameters (such as SPE cartridge, injection volume and washing step) were optimized. The optimized SPE-LC–Q-TOF–MS method showed good recovery (82.0–102.8%), linearity (*r*^2^ ≥ 0.9991) and precision (0.83–4.83%). The limit of detections (LODs) for the five analytes ranged from 0.34 to 0.76 ng mL^−1^. The validated method was successfully applied for determination of the analytes in the neem leaves and seeds from different locations and a neem formulation. The online SPE-LC–Q-TOF–MS method was found to be a simple, precise and accurate and can be used as a powerful tool for quality control of neem extracts or its formulations.

**Electronic supplementary material:**

The online version of this article (10.1186/s13065-018-0453-y) contains supplementary material, which is available to authorized users.

## Introduction

Neem (*Azadirachta indica*) belongs to the family Meliaceae that is well-known for its insecticidal and biomedical properties [1]. For example, the leaf and seed extracts are applied to treat infestations of lice, a common use in Europe [2]. The neem extract has been found to possess many bioactive properties, such as antioxidant [3], antiviral [4], antitumor [5], antimalarial [6] as well as antifungal [7] activities. The neem extracts are rich in limonoids, which could be responsible for these widespread activities. Among the limonoids, azadirachtin A and its structural analogues are considered as active compounds in natural bio-pesticides, which are also considered to be biodegradable and environmental safety [8].

The amounts of azadirachtins in neem extracts varies in different parts of the plant, providing a variety of pesticidal activities [9]. The neem based formulations may show the wide variability in the content of the active principles, which affects the efficacy, reliability and quality of the products [10]. Therefore, each azadirachtin compound and its exact concentration are important for the quality control of neem extracts or its formulations. The analytical methods in relation to neem metabolites have been developed, such as enzyme-linked immunosorbent assay [11], high performance liquid chromatography (HPLC) [12–14] and liquid chromatography–mass spectrometry (LC–MS) [15, 16]. The HPLC methods often applied in the quantification of azadirachtins, but its absorption wavelength is at very short zone where the solvents peaks absorb strongly [9]. Furthermore, the interfering components can not be easily removed by simple purification methods.

Online solid-phase extraction (online-SPE) method could be a good choice for sample purification. Online-SPE technology is a fully automated method for sample preparation that allows direct injection of samples for analysis [17]. This procedure is not only faster than manual samples pre-treatment, but can improve reproducibility [18]. Online-SPE coupled with LC–MS has been successfully applied for qualitative and quantitative analysis of the chemical constituents in plant samples [19].

**Fig. 1:**
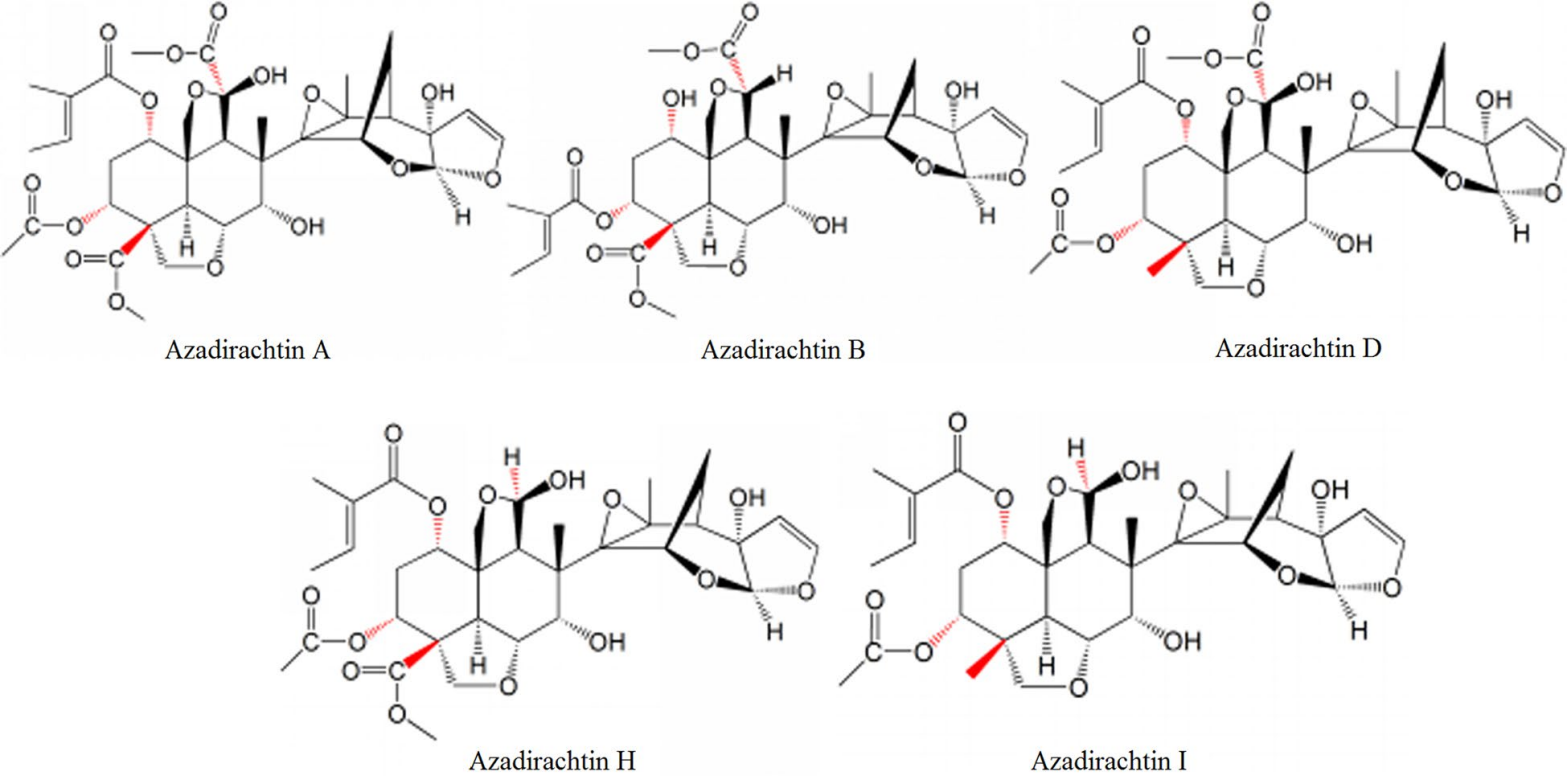
Chemical structures of the five investigated azadirachtins A, B, D, H and I

Online SPE coupled with liquid chromatography/quadrupole-time-of-flight tandem mass spectrometry (LC–Q-TOF–MS) is a powerful strategy, that could be used for the analysis of five azadirachtins (Fig. 1), including azadirachtin A (AZ-A), azadirachtin B (AZ-B), azadirachtin D (AZ-D), azadirachtin H (AZ-H) and azadirachtin I (AZ-I). The aim of this study was to develop and validate a fully automated online SPE-LC–Q-TOF–MS method for determination of the five azadirachtins in the leaf and seed extracts of *A. indica*.

## Materials and methods

### Plant materials and chemicals

Different seeds (No. S1, No. S2 and No. S3) of *A. indica* were collected from Yuanmou County (101°51′E, 25°40′N), Yuanjiang County (102°02′E, 23°61′N), and Jianshui County (102°86′E, 23°22′N), Yunnan Province, China, respectively, in August 2017. Neem leaves (No. L1 and L2) were collected from Yuanjiang County (102°02′E, 23°61′N), Yunnan Province, China. The neem leaves were air dried under shade, ground to powder, and stored at − 20 °C. The neem seeds were manually removed from the fruits and ground in an iced mortar with liquid nitrogen.

HPLC-grade methanol (MeOH) and acetonitrile (ACN) were obtained from Fisher Scientific (Fair Lawn, NJ, USA). Sodium acetate was purchased from CNW Technologies GmbH (Dusseldorf, Germany). Standards of azadirachtins A, B, D, H and I were prepared in our laboratory with purity greater than 95% using HPLC method [20]. Neem pesticide formulation (0.6% azadirachtin EC) was purchased from the market.

### Sample preparation

Sample extraction was based on the previous study with some modifications [21]. A portion (0.10 g) of well-homogenized powdered leaves or seeds was weighted in a 40 mL glass bottle. After adding 20 mL of 70% (v/v) acetonitrile to the bottle, the mixture was extracted in an ultrasonic cleaning bath (KQ-800E, 800W, Kunshan Ultrasonic Instruments Co., Ltd., Kunshan, China) for 30 min. As to the seed samples, the extraction step was repeated twice. The leaf samples were extracted only once. After centrifugation at 5000 rpm for 5 min, 1 mL of supernatant was transferred into a 10 mL volumetric flask and diluted to volume with water.

The neem pesticide formulation (50 μL) was dissolved in 10 mL of acetonitrile and extracted by ultrasonic assisted method for 5 min. One mL of sample was transferred into a 10 mL volumetric flask and diluted to volume with water. The final sample solution was passed through a syringe filter membrane (0.22 µm) before injection.

### Online SPE-LC system conditions

Online SPE-LC separation was performed on a Symbiosis™ Pico system (Spark Holland, Emmen, Netherlands) equipped with an auto-sampler with a 100 µL sample loop, a high pressure dispenser (HPD) module and two binary LC pumps. SPE cartridges were used for sample concentration and cleanup. Three different SPE cartridges, including HySphere™ C18 HD (10 × 2 mm i.d., 7 μm), HySphere™ Resin SH (10 × 2 mm i.d., 15–25 μm) and HySphere™ Resin GP (10 × 2 mm i.d., 10–12 μm) were tested. Sample was injected and loaded onto the cartridge for online sample clean-up and concentration. Different sample volumes (5, 10, 20, 35 and 50 µL) were tested. The flow rate of loading phase was maintained at 700 µL min^−1^ and kept for 1 min. All the tests were carried out in triplicate. The loading phase selected was 10% MeOH. High pressure dispenser (HPD) mode with peak focusing was selected. The SPE parameters were listed in Table 1.

**Table 1:** Online solid phase extraction (SPE) operating procedures

Step	Operation	Solvent	Flow rate (µL min^−1^)	Volume (µL)
1	Activation	MeOH	5000	1000
2	Equilibration	H_2_O	5000	1000
3	Loading SPE	10:90 MeOH/H_2_O	700	700
4	Washing SPE	30:70 MeOH/H_2_O	5000	1000
5	Elution	MeOH	150	300

The washing step was optimized to remove interferences from the SPE column. The optimized washing step was carried out using spiked standard samples, including AZ-A (375 ng mL^−1^), AZ-B (75 ng mL^−1^), AZ-D (50 ng mL^−1^), AZ-H (25 ng mL^−1^) and AZ-I (12.5 ng mL^−1^). After the washing step, the target analytes were eluted from the SPE cartridge, followed by remixing with the LC eluent, resulting in a total flow rate of 400 μL min^−1^ onto an analytical column. The chromatographic separation was performed on a C18 column (150 mm × 2.1 mm i.d., 3.5 µm, Zorbax Eclipse XDB, Agilent USA) at 25 °C. The LC mobile phase consisted of H_2_O (solvent A) and ACN (solvent B) with 10 μM sodium acetate, respectively. The gradient program was as follows: 0–2 min, 10% B; 2–2.08 min, 10–50% B; 2.08–2.5 min, 50–40% B; 2.5–7 min, 40% B; 7–7.08 min, 40–90% B; 7.08–10 min, 90% B; 10–10.08 min, 90–10% B; 10.08–12 min, 10% B. The flow rate was set at 0.25 mL min^−1^ in the first 2 min, then the flow rate was set at 0.4 mL min^−1^.

### MS spectrometry

The quantitative analysis of the five analytes was carried out using an Agilent 6540 Q-TOF–MS system (Agilent Technologies, Santa Clara, CA, USA) equipped with a jet stream ESI interface. The MS data were obtained in a MS scan mode. Mass spectra were recorded from *m*/*z* 50 to 800 in positive ionization mode. The optimized mass analysis conditions were as follows: drying gas (N_2_) flow rate, 10 L min^−1^; drying gas temperature, 350 °C; nebulizer, 310 kPa; sheath gas temperature, 250 °C; capillary voltage, 4000 V; fragmentor voltage, 140 V; nozzle voltage, 500 V; octopole RF voltage, 750 V. All the operations and data analysis were controlled using an integrated software system including Symbiosis Pico in Analyst™ version 1.2.00 (Spark Holland) and MassHunter B.04.00 software (Agilent Technologies, USA).

### Calibration curves and limits of detection

Stock solutions of the five analytes (AZ-A AZ-B AZ-D, AZ-H and AZ-I) were prepared in methanol at concentrations of 3000, 1200, 800, 400 and 200 μg mL^−1^, respectively. Working solutions were prepared by diluting aliquots of stock solutions with 10% methanol. The desired calibration concentrations were obtained using two-fold serial dilutions. The calibration curves for the five analytes were constructed by plotting the peak area (EIC signal of MS) against the concentration at least seven concentrations. According to ICH guideline [22], the limit of detection (LOD) and limit of quantification (LOQ) were calculated as 3.3σ/S and 10σ/S, where S is the slope of the calibration plot and σ is the standard deviation of the response.

### Accuracy, precision and repeatability

The accuracy of the method was calculated by spike-recovery experiments, which was evaluated by adding three concentration levels (low, middle and high) of standard solutions into the seed and leaf samples. The samples of each level were spiked in triplicates. Then the mixtures were analyzed according to the developed method.

Intra- and inter-day variations were used to test the precision of the proposed method. For intra-day precision, the solution of seed sample was analyzed for six replicates in 1 day. For inter-day test, the seed sample was analyzed in duplicates for 3 days consecutively. Six independent samples (sample No. S2) were analyzed in parallel for the measurement of repeatability. All of these treatments were judged with relative standard deviation (RSD).

### Method application

The final developed method has been applied for the identification and simultaneous quantification of five azadirachtins in the seeds and leaves of neem, and a commercial product of neem pesticide formulation. The identification of the five analytes was performed by comparing accurate mass and their retention times with those of standard compounds.

### Statistical analysis

Statistical significance was carried out applying one-way ANOVA followed by Duncan’s test at *p* = 0.05, using SPSS Statistics version 20.0 (SPSS Inc., Chicago, IL, USA). Origin Pro software (Version: 8.5.0 SR1) was used to fit the data and draw the figures.

## Results and discussion

### Optimization of LC–Q-TOF–MS conditions

**Fig. 2:**
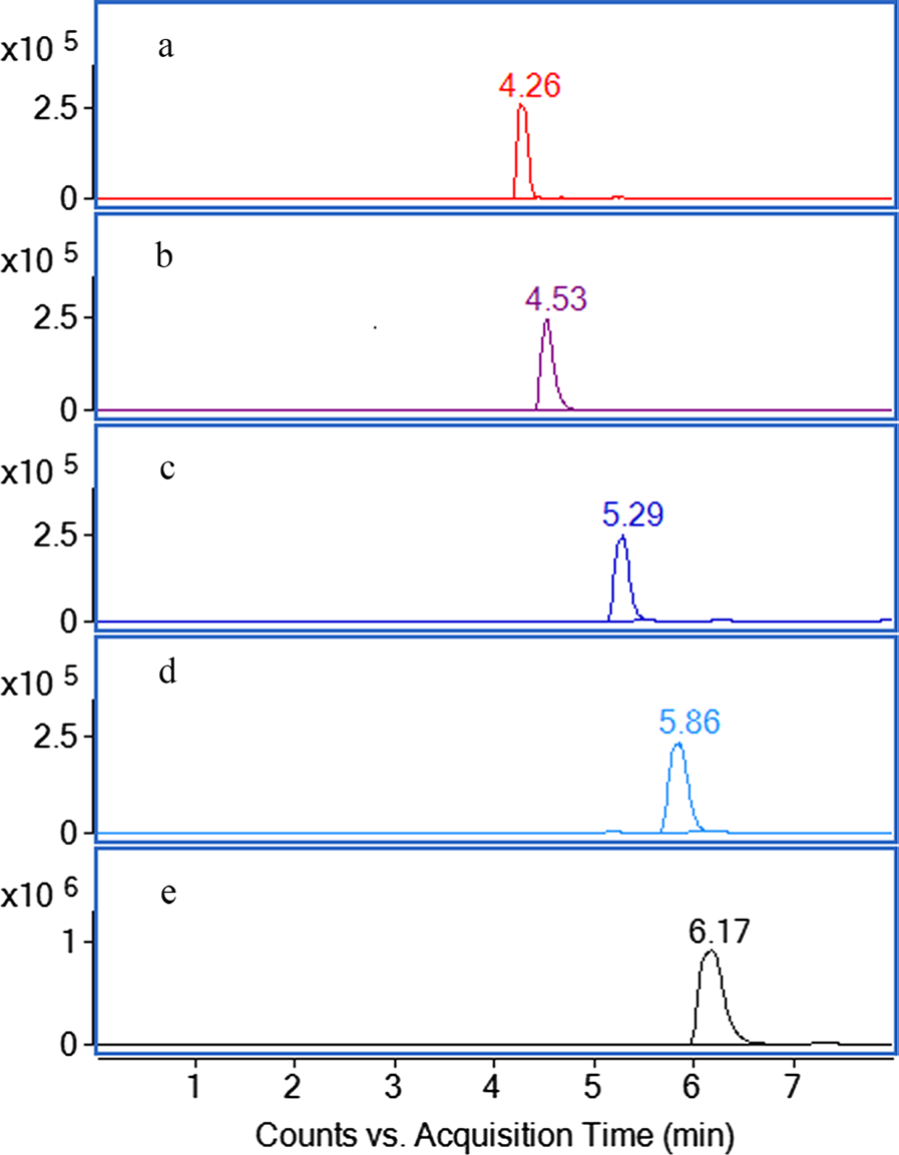
Liquid chromatography/quadrupole-time-of-flight mass spectrometry (LC–Q-TOF–MS) extraction ion current (EIC) of five standards. Peaks a, b, c, d and e correspond to azadirachtins I, H, D, A, and B

Different mobile phase compositions such as acetonitrile–water and methanol–water solvents were tested. To obtain stable product ions and high responses, 10 μM sodium acetate was added into the mobile phase. The gradient mode of acetonitrile–water solvents as the mobile phase, were better than methanol–water for a satisfactory MS response and chromatographic resolution. The positive ionization mode was selected for the quantification and identification of the five analytes for its most intense response. A good separation of all the five analysts were obtained in a short runtime (8 min). Furthermore, MS parameters including fragmentor voltage and drying gas temperature were optimized. The extraction ion current (EIC) chromatograms of the five analytes are shown in Fig. 2.

### Optimization of online-SPE conditions

#### Recovery of online SPE cartridges

**Fig. 3:**
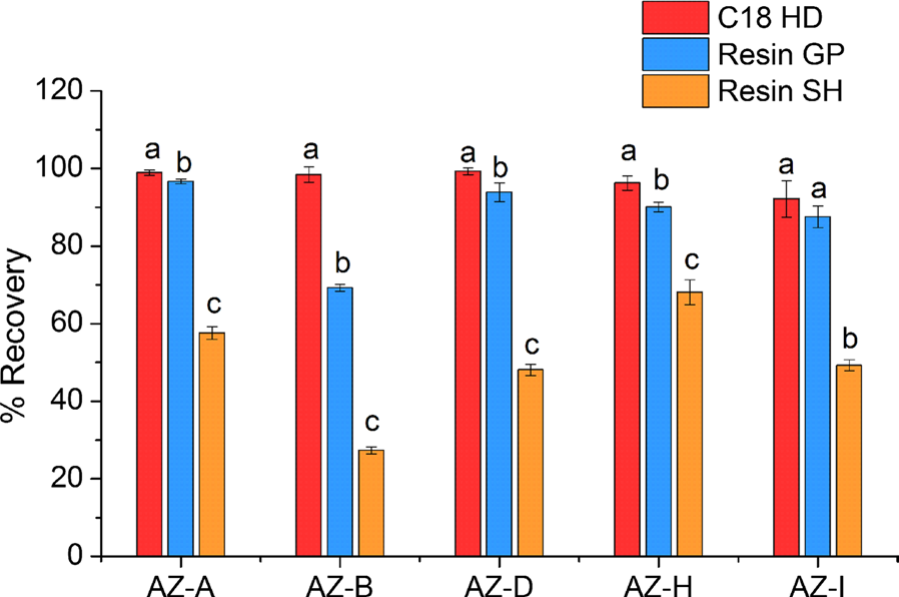
Comparison of recoveries for the five analytes, including azadirachtin A, B, D, H and I, based on three type of SPE cartridges. Standard deviation represented by error bars (n = 3)

The choice of SPE adsorbent material is an important factor for obtaining high recovery [23]. The sample purification step was necessary to remove the possible interference for the determination of azadirachtins using LC or LC–MS [24, 25]. The azadirachtins possess the characteristics of medium polarity, and therefore medium-polar SPE cartridges were considered. Three different SPE cartridges were evaluated. The results showed that HySphere™ C18 HD cartridge provided a good recovery and reproducibility (Fig. 3). Thus, the HySphere C18 HD cartridge was selected in this study. In our laboratory, HySphere C18 HD cartridges could be used repeatedly at least ten times by washing with 1 mL of methanol followed aqueous solvents each time. This means a decrease in the cost and low consumption of organic solvents.

#### Injection volume

**Fig. 4:**
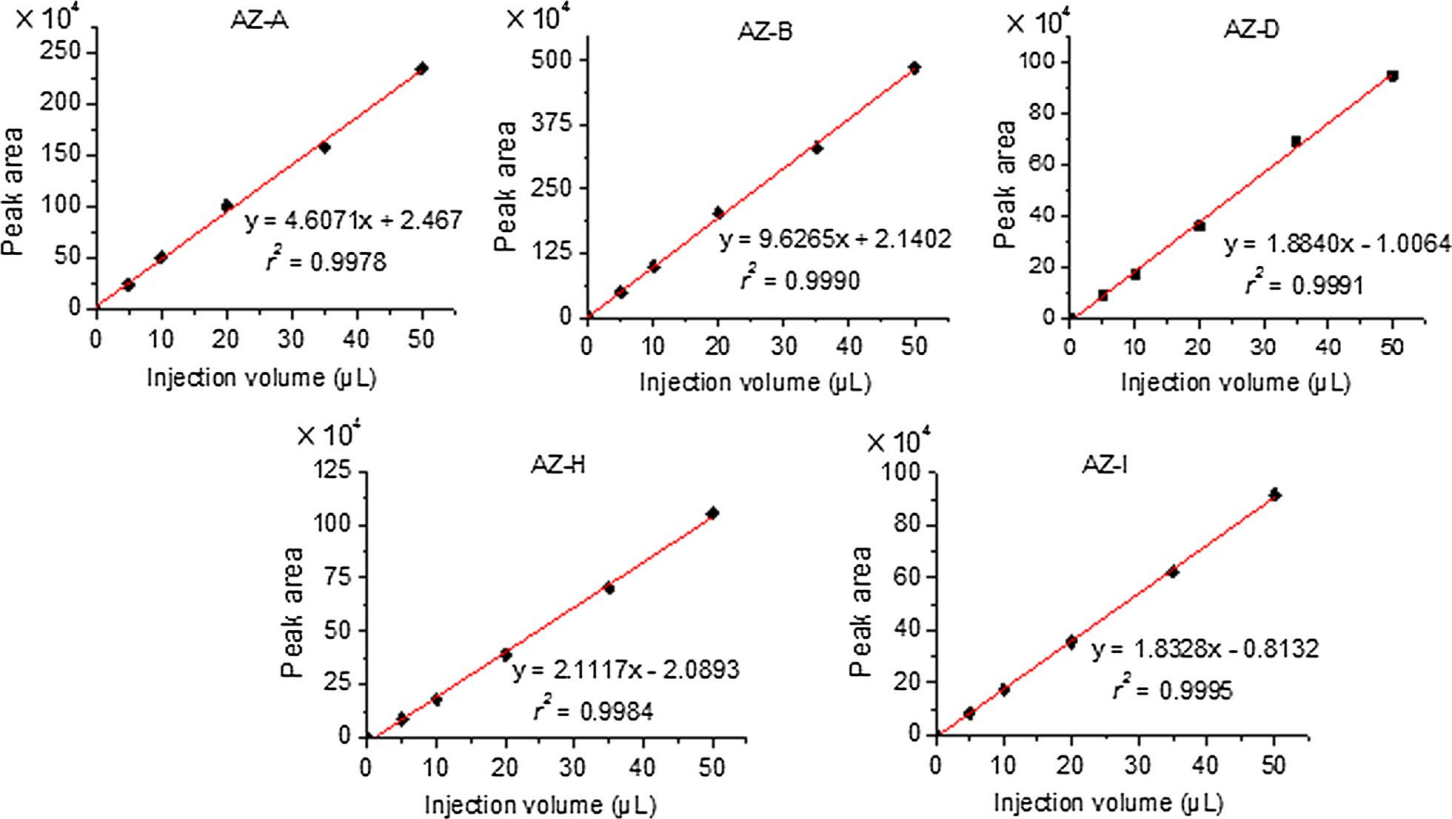
Linear curves of injection volumes and peak areas of the five azadirachtins

The amount of sample loaded on SPE cartridge affects the sensitivity of the analytical method [26]. The effect of sample injection volume on peak area of the analytes was investigated. Peak areas were plotted versus injection volumes to produce five linear curves (Fig. 4). All the curves showed a good linear relationship (*r*^2^ > 0.997). No sample breakthrough was observed within the tested range. The peak areas of the five azadirachtins increased with the increasing of sample volumes, thus the increasing of method sensitivity. To establish a more sensitive method for determination of the five azadirachtins, a relatively larger volume (50 µL) was selected as injection volume using the auto-sampler.

#### Optimization of methanol percentage for loading phase

**Fig. 5:**
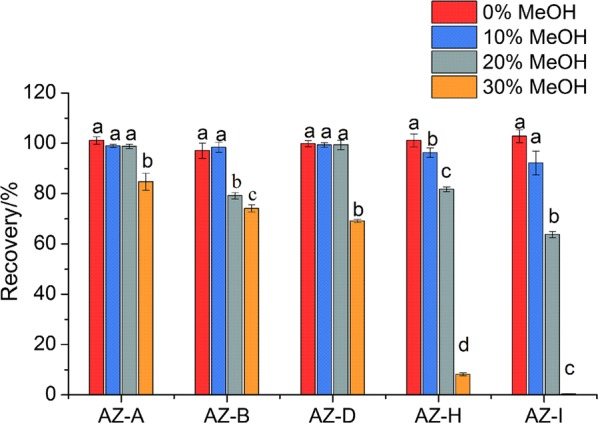
Comparison of the recoveries of five analytes, including azadirachtin A, B, D, H and I, with four different percentages of methanol during loading phase (n = 3)

After injection, the sample was withdrawn into a sample loop and then carried over by the loading phase from a high pressure dispenser (HPD) pump. The composition of methanol in the loading phase effects the recovery of the analytes [27]. The loading phase composition of methanol and water were evaluated in the range of 0–30% with the increment of 10% each time. The satisfactory recoveries were acquired using pure water or 10% MeOH as the loading phase (Fig. 5). Additionally, a significant inverse relation was observed between the methanol percentage of the loading phase and the absolute recoveries of the analytes. The reason for this is the fact that the loading phase with high percentage of methanol could lead to premature column breakthrough.

#### Optimization of methanol percentage for washing phase

**Fig. 6:**
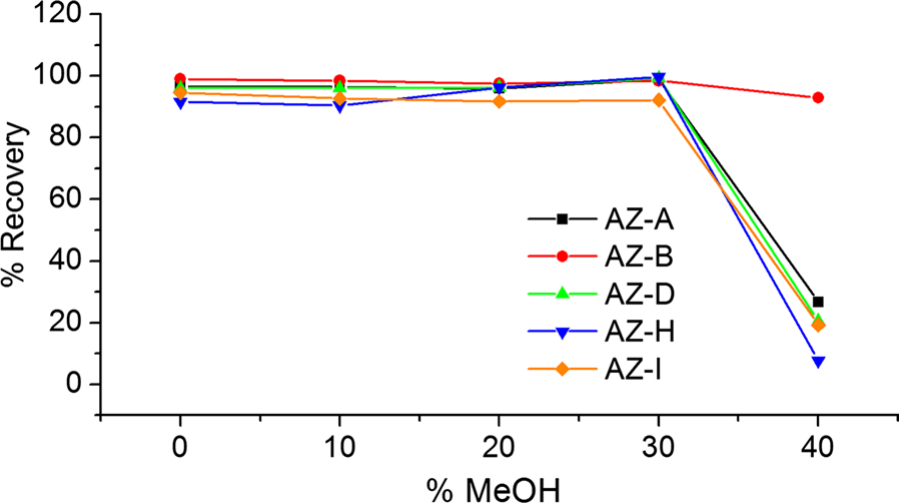
Comparison of the recoveries of five analytes, including azadirachtin A, B, D, H and I, with five different percentages of methanol during washing phase (n = 3)

After sample loading, the composition of washing phase was a significant factor for cleanup step [28]. Five different percentages of methanol were investigated ranging from 0 to 40% with an increment of 10% each time. The recoveries of the analytes were tested for the influence of methanol percentage during the washing phase. The recoveries of all the analytes decreased obviously while the 40% methanol was used (Fig. 6). Therefore, 30% methanol was selected as washing phase as it allowed the best recoveries in the case of remove interferences.

### Method validation

The calibration curves, linear ranges, limits of detection (LOD) and limits of quantification (LOQ) values of five azadirachtins were carried out using an online-SPE-LC–Q-TOF–MS method (Table 2).

**Table 2:** Calibration curves of the five investigated analytes

Compound	Regression equation	*r* ^2^	Range (ng mL^−1^)	LOD (ng mL^−1^)	LOQ (ng mL^−1^)
Azadirachtin A	y = − 861711·x^2^ + 5836597·x + 47030	0.9992	23.44–3000	0.45	1.35
Azadirachtin B	y = − 2305665·x^2^ + 12766095·x − 121354	0.9992	18.75–1200	0.34	1.04
Azadirachtin D	y = 591578·x^2^ + 4267977·x − 754	0.9998	3.12–800	0.76	2.30
Azadirachtin H	y = 13670508·x^2^ + 5608355·x + 12303	0.9991	3.12–400	0.42	1.25
Azadirachtin I	y = 11915995·x^2^ + 3434963·x + 4502	0.9996	3.12–200	0.46	1.40

The correlation coefficient values (*r*^2^ ≥ 0.9991) demonstrated good correlation with given concentration ranges. The external calibration curves were constructed by using polynomial regression. The sensitivity expressed as LOD and LOQ were less than 0.76 and 2.30 ng mL^−1^, respectively.

The RSD values of the peak areas of the five analytes were with the range of 2.12–4.55%. The results for intra-day (0.83–4.62%) and the inter-day (1.67–4.83%) showed good precision. Meanwhile, the retention time variations (RSD) were less than 0.11 and 0.26%, respectively (Table 3).

**Table 3:** Repeatability and precision of the five analytes

Analytes	Repeatability (RSD, n = 6) %	Intra-day		Inter-day	
		(RSD, n = 6)%		(RSD, n = 6)%	
		Retention time	Peak area	Retention time	Peak area
Azadirachtin A	3.93	0.04	4.62	0.13	4.83
Azadirachtin B	3.02	0.11	0.83	0.20	1.67
Azadirachtin D	2.12	0.10	1.84	0.21	2.34
Azadirachtin H	2.32	0.10	2.10	0.26	2.77
Azadirachtin I	4.55	0.09	2.55	0.18	3.44

Good recoveries of 82.0–102.8% with RSD of 0.04–8.11% were obtained in this study (Table 4).

**Table 4:** Recovery test of the five azadirachtins in the neem samples (n = 3)

Compound	Seed			Leaf		
	Spiked (µg)	Recovery (%)	RSD (%)	Spiked (µg)	Recovery (%)	RSD (%)
Azadirachtin A	150	99.9	0.04	70	100.9	0.53
	300	86.1	3.23	140	87.9	4.59
	600	93.5	5.62	280	83.3	0.56
Azadirachtin B	25	93.4	8.11	5	95.9	6.19
	50	87.8	2.79	10	98.9	3.40
	100	83.1	1.38	20	93.4	1.89
Azadirachtin D	14	85.6	3.39	0.7	93.8	7.03
	28	91.9	3.81	1.4	95.7	1.06
	56	97.2	1.02	2.8	83.9	3.71
Azadirachtin H	10	90.4	4.73	2	102.8	6.83
	20	82.0	3.25	4	92.1	2.26
	40	83.4	1.80	8	88.2	1.34
Azadirachtin I	4.5	102.8	3.60	1.25	99.5	3.35
	9	90.6	3.27	2.5	95.9	2.55
	18	94.0	3.93	5	85.7	1.43

### Analysis of neem samples

The proposed method was successfully applied to analyze the five azadirachtins in *A. indica* from different locations. The contents of the seed and leaf extracts (n = 3) of five azadirachtins and also the neem formulation (n = 3) are shown in Table 5.

**Table 5:** Contents of azadirachtin A, B, D, H and I in different neem samples (n = 3)

Name	Sample no.	Mean contents (µg g^−1^) ± S.D (standard deviation)				
		AZ-I	AZ-H	AZ-D	AZ-A	AZ-B
Seed	S1	47.6 ± 1.4	110.4 ± 1.8	229.2 ± 3.5	3862.9 ± 7.7	578.8 ± 2.1
	S2	98.9 ± 2.0	201.7 ± 8.9	760.9 ± 6.5	4852.1 ± 234.0	952.8 ± 40.5
	S3	94.7 ± 5.1	205.7 ± 0.6	510.9 ± 18.4	4669.7 ± 58.6	900.5 ± 12.1
Leaf	L1	7.4 ± 0.4	60.3 ± 0.6	5.4 ± 0.4	130.2 ± 0.9	10.7 ± 0.5
	L2	29.1 ± 0.6	173.5 ± 1.8	27.9 ± 0.5	969.9 ± 7.9	64.5 ± 0.2
Neem formulation		178.3 ± 1.8	220.1 ± 3.1	523.0 ± 16.7	2426.1 ± 117.0	678.8 ± 4.5

Because seeds contain the highest concentrations of azadirachtins, most commercial preparations of neem are derived from seed extracts [29]. The commercial products of the neem extracts are usually evaluated by measuring the content of azadirachtin A [30]. Azadirachtins A was the most frequently detected compound in all the neem samples, and the five analytes were also found in the neem formulation (Table 5). According to the previous reports, the neem seeds are considered to be the most abundant source, of which the content of azadirachtin A can reach up to 5419.08 μg g^−1^, whereas the content of azadirachtin A in the neem leaves was 182.42 μg g^−1^ [31]. In this study, the contents of azadirachtin A ranged from 3862.9 to 4852.1 μg g^−1^ in neem seeds. The content of azadirachtin A in the neem leaf extract (sample No. L2) was 969.9 μg g^−1^. The main mass data of the five azadirachtins from neem samples are shown in Additional file 1: Table S1. The contents of azadirachtins in neem seeds were higher than those in neem leaves. Generally, the environmental factors such as climatic and soil conditions can affect chemical composition of the plants. In the previous studies [32, 33], wide variations have been found in azadirachtin contents of neem seeds from different provenances and also between individual trees of a particular location. It has been proved that the variations in azadirachtins are attributed to individual genetic differences among neem trees other than climatic factors [33]. Additionally, azadirachtin is very labile when exposed to air, moisture and sunlight. Its instability to UV radiation may also affect the percentage of azadirachtin present in neem seeds or leaves [25].

Neem extracts and pure azadirachtin are one of the most significant insecticides authorized for organic farming crop protection in many countries, which are used to control agricultural pests [34]. An analysis of *A. indica* is very important as quality control, since the primary interest is its insecticide activity [35]. Therefore, the selected five azadirachtins found in all the neem seeds were suitable as marker compounds for quality control of the neem extracts. Furthermore, these results indicate the proposed method is a useful tool for determination of the five markers in *A. indica* from different locations. Further studies on the qualitative and quantitative analysis of the other limonoids found in traces and existed synergy among constituents in the extracts of *A. indica* are needed.

## Conclusions

A fully automated online SPE-LC–Q-TOF–MS method was developed for the simultaneous determination of five azadirachtins in the seed and leaf extracts of *A. indica*. The online SPE-LC system was able to provide high throughput sample preparation, good reproducibility and large volume sample injection. The Q-TOF–MS system enabled the identification of the five azadirachtins with high selectivity. The method was validated and found to be precise, accurate and sensitive. The proposed method was successful applied to quantify the five azadirachtins in different neem samples and a neem formulation. The online SPE-LC–Q-TOF–MS method can be used as a tool for quality control of neem plant or its formulations.

## Additional file

**Additional file 1: Table S1.** Mass data of the five azadirachtins from neem samples by online-SPE-LC-Q-TOF–MS.
