# Supplementary material for: Simultaneous determination of five azadirachtins in the seed and leaf extracts of Azadirachta indica by automated online solid-phase extraction coupled with LC–Q-TOF–MS
Source: Chem Cent J. 2018 Jul 19;12:85. doi: 10.1186/s13065-018-0453-y (PMC6053346; doi:10.1186/s13065-018-0453-y)
Supplement: Supplementary file 1 — Additional file 1: Table S1. Mass data of the five azadirachtins from neem samples by online-SPE-LC-Q-TOF–MS. [file 13065_2018_453_MOESM1_ESM.docx]

**Table** S**1** Mass data of the five azadirachtins from neem samples by online-SPE- LC-Q-TOF-MS

| No. | RT  (min ) | Exact  mass (*m/z*) | Theoretical  [M+Na]^+^( *m/z*) | Observed  [M+Na]^+^( *m/z*) | Error  (ppm) | Formula | Identification |
| --- | --- | --- | --- | --- | --- | --- | --- |
| 1 | 4.25 | 618.2676 | 641.2568 | 641.2567 | 0.24 | C_32_H_42_O_12_ | Azadirachtin I |
| 2 | 4.53 | 662.2575 | 685.2467 | 685.2463 | 0.57 | C_33_H_42_O_14_ | Azadirachtin H |
| 3 | 5.29 | 676.2731 | 699.2623 | 699.2610 | 1.96 | C_34_H_44_O_14_ | Azadirachtin D |
| 4 | 5.86 | 720.2629 | 743.2522 | 743.2510 | 1.61 | C_35_H_44_O_16_ | Azadirachtin A |
| 5 | 6.15 | 662.2575 | 685.2467 | 685.2473 | -0.94 | C_33_H_42_O_14_ | Azadirachtin B |
